# The Contribution of Oxidative Stress to *NF1*-Altered Tumors

**DOI:** 10.3390/antiox12081557

**Published:** 2023-08-04

**Authors:** Elisabetta Kuhn, Federica Natacci, Massimo Corbo, Luigi Pisani, Stefano Ferrero, Gaetano Bulfamante, Donatella Gambini

**Affiliations:** 1 Department of Biomedical, Surgical and Dental Sciences, University of Milan, 20122 Milan, Italy; stefano.ferrero@unimi.it (S.F.); gaetano.bulfamante@unimi.it (G.B.); 2 Pathology Unit, Foundation IRCCS Ca’ Granda Ospedale Maggiore Policlinico, 20122 Milan, Italy; 3 Medical Genetics Unit, Foundation IRCCS Ca’ Granda Ospedale Maggiore Policlinico, 20122 Milan, Italy; federica.natacci@policlinico.mi.it; 4 Department of Neurorehabilitation Sciences, Casa di Cura Igea, 20144 Milan, Italy; m.corbo@casadicuraigea.it (M.C.); l.pisani@casadicuraigea.it (L.P.); d.gambini@casadicuraigea.it (D.G.); 5 Human Pathology and Molecular Pathology, TOMA Advanced Biomedical Assays S.p.A., 21052 Busto Arsizio, Italy

**Keywords:** neurofibromatosis, *NF1*, pathogenesis, neurofibromin, oxidative stress, ROS, therapy

## Abstract

The neurofibromatosis-1 gene (*NF1*) was initially characterized because its germline mutation is responsible for an inherited syndromic disease predisposing tumor development, in particular neurofibromas but also various malignancies. Recently, large-scale tumor sequencing efforts have demonstrated *NF1* as one of the most frequently mutated genes in human cancer, being mutated in approximately 5–10% of all tumors, especially in malignant peripheral nerve sheath tumors and different skin tumors. *NF1* acts as a tumor suppressor gene that encodes neurofibromin, a large protein that controls neoplastic transformation through several molecular mechanisms. On the other hand, neurofibromin loss due to *NF1* biallelic inactivation induces tumorigenic hyperactivation of Ras and mTOR signaling pathways. Moreover, neurofibromin controls actin cytoskeleton structure and the metaphase–anaphase transition. Consequently, neurofibromin deficiency favors cell mobility and proliferation as well as chromosomal instability and aneuploidy, respectively. Growing evidence supports the role of oxidative stress in *NF1*-related tumorigenesis. Neurofibromin loss induces oxidative stress both directly and through Ras and mTOR signaling activation. Notably, innovative therapeutic approaches explore drug combinations that further increase reactive oxygen species to boost the oxidative unbalance of *NF1*-altered cancer cells. In our paper, we review *NF1*-related tumors and their pathogenesis, highlighting the twofold contribution of oxidative stress, both tumorigenic and therapeutic.

## 1. Introduction

Neurofibromatosis type 1 (*NF1*) is an autosomal dominant genetic trait due to an inherited heterozygous mutation of the *NF1* gene [1]. *NF1* patients have prevalent neurocutaneous involvement associated with variable multisystem complications. Moreover, *NF1* predisposes patients to both benign and malignant tumors, including nerve sheath tumors, gliomas, pheochromocytomas, and others. Unsurprisingly, *NF1* is indeed somatically deficient in a variety of sporadic cancers, accounting for about 5–10% of all human malignancies.

A fine-tuned redox equilibrium between oxidants and antioxidants exists in the human body and is crucial for cell survival [2]. Unbalanced production of reactive oxygen species (ROS) and other free radicals is sustained by microenvironmental and proteotoxic insults, hypoxia, and metabolic anomalies and causes oxidative stress [3]. Persistent sublethal free oxygen radicals may play a pivotal role in the etiopathogenesis of cancer and chronic diseases, such as neurodegenerative and cardiovascular diseases, by inducing cell signaling changes and mutagenic effects [4].

Emerging evidence indicates that *NF1* patients and *NF1*-altered tumors display increased oxidative stress markers. Functionally, *NF1* is a tumor suppressor that encodes a protein that activates the Ras guanosine-triphosphate (GTP)ase, which in turn downregulates Ras by inducing the hydrolysis of GTP bound to Ras (Ras-GTP), its activated form. The tumorigenic inactivation of *NF1* induces an enhancement of the Ras and mechanistic target of rapamycin (mTOR) signaling pathways [5,6]. *NF1* deficiency, both directly and through Ras and mTOR pathways, increases cell oxidative stress, contributing to tumorigenesis [7,8]. Consequently, recent studies explore drug combinations that exploit the oxidative unbalance of cancer cells to further increase ROS as innovative therapeutic approaches, specifically in *NF1*-altered cancers.

In this review, we deal with *NF1*-related tumors and their pathogenesis, highlighting the twofold contribution of oxidative stress, both tumorigenic and therapeutic.

## 2. Neurofibromatosis Type 1

*NF1* (Online Mendelian Inheritance in Man database number 162200), also referred to as Von Recklinghausen disease, is an inherited syndrome that favors the development of tumors and other clinical manifestations. *NF1* was first identified by a German pathologist, Von Recklinghausen, in 1882 [9]. It represents one of the most prevalent human genetic diseases, with an incidence estimated at around 1 in 3000 people globally [9,10], autosomal dominant transmission, and an approximately equal distribution between familial and newly acquired *NF1* mutations [11].

**Figure 1:**
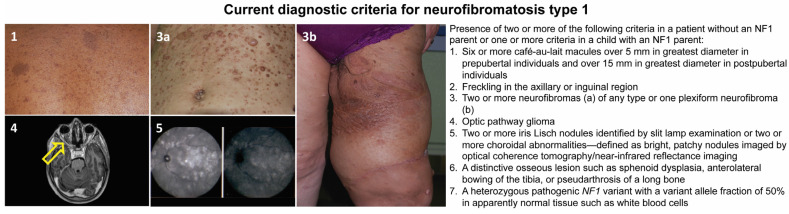
Current diagnostic criteria for neurofibromatosis type 1 and representative pictures of the main clinical findings: café-au-lait macules (**1**); skin neurofibromas of the abdomen (**3a**); plexiform neurofibroma involving the left thigh (**3b**); axial T2-weighted image of the brain and orbits showing left optic nerve glioma (yellow arrow, **4**); and choroidal nodules detected by ocular coherence tomography (**5**).

This disease has a complete penetrance, and clinical signs are progressive and variable throughout life, with some appearing during childhood and others later in adulthood [10]. Although *NF1* is mainly known for its cutaneous manifestations (café-au-lait spots, axillary and inguinal freckling, and cutaneous, subcutaneous, or plexiform neurofibromas), it is a complex and heterogeneous condition that, in addition to the aforementioned features, can involve multiple organs and systems. Affected subjects may, in fact, develop neuropsychological, ophthalmological, orthopedic, neurological, vascular, endocrinological, and oncological complications [11,12,13,14]. Regarding the latter, besides the common neurofibromas, the overall risk of developing cancer is estimated to be 5–15% higher than in the general population, with earlier occurrence and a worse prognosis [14,15,16]. The oncologic predisposition conferred by the *NF1* mutation refers to the development of benign and malignant tumors; in particular, in childhood, the risk is increased for gliomas of the optic pathway, juvenile myelomonocytic leukemia, and embryonal rhabdomyosarcomas [16,17]. In adulthood, the predisposition concerns the development of malignant nerve sheath tumors (MPNSTs), spinal and cerebral tumors, pheochromocytomas, paragangliomas, gastrointestinal stromal tumors (GIST), breast cancer, and subungual glomic tumors [18]. A *NF1* diagnosis can be reached following the international diagnostic criteria shown in Figure 1, recently revised by a panel of international experts [15].

Generally, there is no clear link between a specific genotype and the *NF1* phenotype, showing considerable variability even within the same genetic variant and family. To explain this fact, environmental and epigenetic influencers are called into question [1,19]. Nevertheless, the genotype correlates with the phenotype in the following scenarios: (a) *NF1* complete deletion determines a severe *NF1* phenotype [20]; (b) missense mutations affecting *NF1* codons 844–848 seem to be correlated with a severe presentation [21]; (c) recurrent 3-bp in-frame deletion of exon 17 (c.2970–2972 delAAT, p.Met992del) and missense mutation c.5425C > T (p.Arg1809Cys) are related to pigmentary *NF1* in the absence of cutaneous or surface plexiform neurofibromas [22,23]; and (d) *NF1* missense mutations p.Met1149, p.Arg1276, and p.Lys1423 correlate with a Noonan-like phenotype [24,25].

Many *NF1* patient-derived tumors demonstrate biallelic deficiency of the *NF1* gene [26,27]. Therefore, the acquisition of a secondary somatic hit, which disrupts the wild-type allele with loss of heterozygosity, represents the obligatory molecular event that unveils the neoplastic potential in these patients, but it is not sufficient [26,28]. Inherently, *NF1* must be functionally completely lost to favor tumorigenesis, thereby configurating a tumor suppressor gene [28].

## 3. *NF1*-Altered Sporadic Tumors

**Figure 2:**
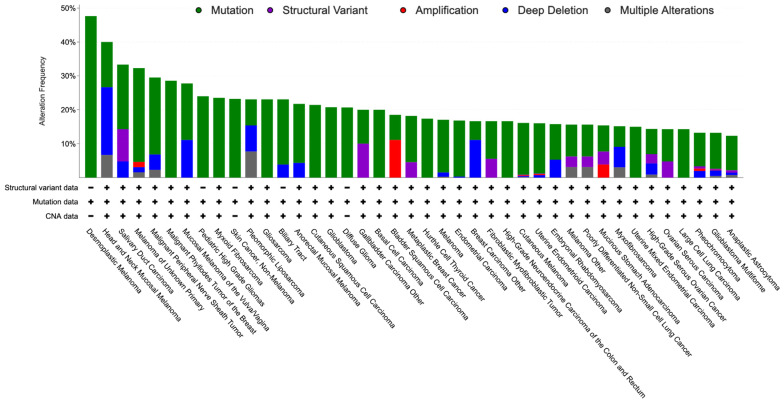
Bar graph showing the percentage of *NF1* alterations (over 10%) in the TCGA by tumor type.

Notably, *NF1* is biallelically inactivated in most tumors developing in *NF1* patients and, based on The Cancer Genome Atlas (TCGA), it is also genetically altered with a frequency up to 82% in a variety of sporadic tumors, particularly MPNST and skin cancers, including desmoplastic melanoma, skin squamous cell and basal cell carcinoma, and cutaneous melanoma, but also, among others, glioblastoma and ovarian high-grade serous carcinoma (Figure 2) [29].

Genetically, *NF1* inactivation is mainly the consequence of sequence mutation, but deep deletion may cause its loss as well. Moreover, *NF1* appears amplified in almost 17% of a special type of breast cancer, i.e., adenoid cystic breast carcinoma [30], and in a significant minority of other specific cancers, including biliary tract, gastric, bladder urothelial, and pancreatic cancers.

Clinically, MPNSTs, as well as being associated with *NF1*, may develop sporadically or be induced by radiotherapy. *NF1* harbors genetic alteration in 82% (18/22) of these cases, supporting its strong driving role also in this clinical setting [31].

Most skin cancers share a common strong mutagen, ultraviolet radiation from sunlight, and are associated with a high tumor burden. Cutaneous melanoma represents a malignant neoplasia of melanocytic cells that appears as a skin-pigmented flat or nodular lesion. *NF1* mutations are identified in 14% of cases and characterize one of the main molecular subtypes [32]. Moreover, desmoplastic melanoma, an aggressive scarring variant typical of old people that constitutes 4% of all skin melanoma, demonstrates *NF1* alterations in up to 55% of cases [33].

Squamous cell carcinoma arises from keratinocytes and is the second most frequent skin cancer. Based on a meta-analysis study, the frequency of *NF1* somatic mutations in this tumor is 28%. However, among the 28 observed mutations in 23 lesions, only nine are likely drivers, whereas the others are missense mutations with unknown biological and oncological effects. Because of the causal effect of ultraviolet radiation, the tumor mutation burden of skin cancers is high, with the consequence that the majority of mutations are non-pathogenetic passengers, making it difficult to identify the actual driver mutations [34].

Similarly, basal cell carcinoma, the most frequent human cancer, derives from keratinocytes due to ultraviolet radiation-induced DNA damage with a high number of mutations. Molecularly, basal cell carcinoma is driven by Sonic Hedgehog pathway activation in the large majority of cases due to *PTCH1*, *SMO*, and *SUFU* somatic mutations in 73%, 20%, and 8% of lesions, respectively [35,36]. Regarding *NF1*, somatic mutations were found in 23% (68 of 293) of samples, but only 30 identified mutations were predicted to be likely oncogenic [36].

Glioblastoma is the most common and aggressive cancer of the central nervous system. *NF1* genetic alterations affect 23% of cases, particularly of the mesenchymal subtype, and are the fourth most frequently altered gene in this cancer, after *EGFR*, *PTEN*, and *TP53* [37,38].

Ovarian high-grade serous carcinoma is an ominous disease molecularly characterized by *TP53* somatic mutations, which are almost universal in this neoplasia and the main malignant driver [39,40]. In this cancer, only nine other genes appear to be recurrently mutated, including *NF1* together with *BRCA1*, *BRCA2,* and *RB* [40]. Altogether, *NF1* appeared genetically altered in 14.6% of cases, with mutations and deep deletions found in 8.3% and 6.3%, respectively [41].

## 4. Molecular Genetics of *NF1* Inactivation

*NF1* is a large tumor suppressor gene, covering a 383 kilobase genomic DNA region, sited at chromosome 17q11.2, comprising 61 exons, and encoding for neurofibromin [1,42]. The identification of *NF1* genetic variants is arduous due to the relevant length of the gene, high mutability, lack of mutation hotspots or clustering, common deep intronic variants, the presence of various pseudogenes, and heterogeneous cell populations, particularly in benign tumors [43,44,45,46,47,48,49].

Regardless, identified pathogenetic mutations are the most diverse; the majority are splicing site, missense, or nonsense mutations that interfere with the normal length of the transcript, causing early protein truncation. Therefore, the most frequent mutations affecting *NF1*, both germinally and somatically, lead to loss-of-function neurofibromin [46,47]. On the other hand, approximately 5% of *NF1* patients harbor a complete gene deletion [50].

Commonly, to express oncogenic potential, a tumor suppressor gene must receive a second genetic hit that gives it an oncogenic advantage. The genetic mechanism that initiates tumorigenesis in *NF1*-related tumors, both syndromic and sporadic, is a somatic inactivation of *NF1*. It superimposes either a congenital inherited germline mutation or a somatically acquired *NF1* loss-of-function event. Of note, congenital *Nf1* homozygous null mutations in mice are lethal as a consequence of severe heart malformations [51,52].

Loss of heterozygosity (LOH), by definition, a wild-type allele loss at a heterozygous locus resulting in a consequent allelic imbalance, is the preferential inactivation mechanism of tumor suppressor genes. LOH can derive from partial chromosomal deletion, complete chromosomal loss, or mitotic recombination [53,54]. In *NF1*-related tumors, copy-neutral LOH with retention of normal copy number is the prevalent molecular somatic second hit [53]. It is thought to derive from deletion with concurrent chromosome duplication or, more often, mitotic recombination [54].

Finally, some tumors with wild-type *NF1* may also display *NF1* protein reduction by posttranscriptional inhibition, such as microRNA silencing and proteasome-mediated *NF1* protein degradation, but it is unlikely by epigenetic silencing through promoter methylation [55,56,57].

## 5. *NF1*-Related Tumorigenic Mechanisms

Neurofibromin is a very large, ubiquitous protein, particularly highly expressed in the cells of the central and peripheral nervous systems, including neurons, astrocytes, oligodendrocytes, and Schwann cells [42]. The *NF1* gene produces distinct isoforms by alternative splicing, the principal and better known are neurofibromin types I, II, III, and IV [58].

### Neurofibromin Cellular Functions

Neurofibromin type I acts as a Ras regulator, mainly in the brain. Neurofibromin type II, which contains exon 23a, contributes to learning and memory abilities and is expressed in Schwann cells [58]. Neurofibromin type III has an exon 48a insertion, while neurofibromin type IV displays exons 23a and 48a; both are expressed in cardiac and skeletal muscles and contribute to their development.

**Figure 3:**
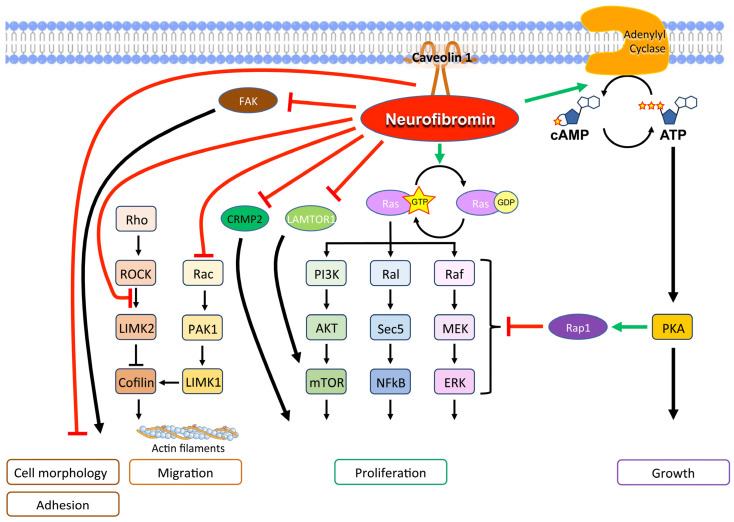
Main molecular signals of neurofibromin with a tumor suppression function.

Physiologically, neurofibromin is a GTPase-activating protein (GAP), which downregulates Ras family proteins, including H-Ras, K-Ras, N-Ras, M-Ras, R-Ras1, and R-Ras2, differentially in various cell types. [5]. Specifically, central exons between 20 and 27a encode the GAP-related domain that stimulates GTPase, which in turn hydrolyzes GTP bound to Ras, converting Ras-GTP to Ras-GDP, its inactivated form [59]. Not only that, neurofibromin stimulates the enzyme adenylyl cyclase, which inhibits the Ras pathway through Rap1, its potent antagonist [60,61]. Therefore, *NF1*-mutated cells demonstrate hyperactivation of the Ras/mitogen-activated protein kinase (MAPK) signaling pathway, a major driver of cancer (Figure 3).

Also, the mTOR pathway is activated in *NF1*-related tumors, both as a consequence of Ras pathway activation and since functional neurofibromin interacts with late endosomal/lysosomal adaptor, MAPK and mTOR activator 1 (LAMTOR1), inhibiting mTOR complex 1 signaling [6,62].

In addition, neurofibromin forms a complex with caveolin-1 that controls cell differentiation and proliferation by interfering with the PI3K/Akt pathways [63].

Neurofibromin is crucial in controlling cytoskeletal structure and contributes to cell adhesion and mobility [64]. In particular, the Rac1/Pak1/LIMK1/cofilin and Rho/ROCK/LIMK2/cofilin pathways, which control the dynamic reorganization and turnover of actin filaments, are negatively regulated by neurofibromin [64,65]. Furthermore, the neurofibromin C-terminal domain directly interacts with the focal adhesion kinase (FAK), one of the main components of the focal adhesion complexes, which play a role in cell-extracellular matrix adhesion [66]. Neurofibromin-deficiency-determined cell morphology changes and an elongated cell appearance, which were associated with increased actin and FAK expression as well as increased cell growth and adhesion [66,67]. These findings support a multifaceted role of neurofibromin in actin cytoskeleton reorganization, cell motility, and proliferation [65,66].

Neurofibromin likely also controls cell motility by inhibiting epithelial–mesenchymal transition and the expression of related transcription factors Slug, Snail, Twist, Zeb1, and Zeb2 [68].

Neurofibromin participated in the spindle assembly checkpoint, causing mitotic arrest in response to spindle damage [69]. In this way, it controls the metaphase–anaphase transition. Moreover, neurofibromin is localized to the mitotic spindle and participates in chromosomal alignment during metaphase, allowing appropriate chromosome congression during mitosis [58]. As a consequence, neurofibromin deficiency favors chromosomal instability and aneuploidy [70].

In neurons, neurofibromin binds collapsin response mediator protein-2 (CRMP2), a protein involved in axonal outgrowth, and promotes neurite outgrowth by preventing CRMP2 from being phosphorylated by cyclin-dependent kinase 5 (Cdk5) [71]. A lack of neurofibromin increases CRMP2 phosphorylation which, in turn, promotes glioblastoma cell proliferation and survival [72].

## 6. Oxidative Stress and Cancer

A balance between reducing and oxidizing (redox) mechanisms is essential for maintaining cellular homeostasis and human health, and many reactions contribute to achieving a redox balance. However, when the oxidant burden prevails, with ROS exceeding antioxidants, a condition known as oxidative stress develops. Oxidative stress has been linked to many pathological conditions, such as cardiovascular, neurodegenerative, metabolic, and inflammatory diseases, as well as cancer [73].

A redox balance is the best condition for a proper cellular metabolism, but oxidative stress can act as a two-faced Janus, especially for cancer. On the one hand, increased ROSare recognized as a risk factor for carcinogenesis, producing DNA damage, but on the other hand, an excess of oxidant species can be cytotoxic.

Many studies have been conducted regarding the link between oxidative stress and cancer at different stages of development since ROS are relevant products of the cancer cell’s metabolism. A higher amount of ROS is characteristic of tumoral versus normal cells, a mechanism sustained by different factors. An increased ROS production by mitochondrial nicotinamide adenine dinucleotide phosphate (NADPH) oxidase and 5-lipoxygenase has been associated, respectively, with hypoxia or defective electron transport chains, hyperproliferation or centrosome anomalies, and cell mobility, while an increased production of H_2_O_2_ inside the endoplasmic reticulum seems related to protein folding [74]. Several oncogenes are involved in the increased ROS production by cancer cells. Among these, Ras, rac1, STAT3, BCL2, and MYC are described as being able to interfere with the mitochondrial metabolism, resulting in the activation of enzymes involved in the redox balance, such as NADPH oxidase [74]. The increase in ROS in tumoral cells can produce a favorable cellular environment, supporting proliferation and survival, by downregulating the activities of some phosphatases, such as MAPK phosphatases, PTEN, and protein tyrosine phosphatases, resulting in the downstream activation of a pathway signaling cascade involving PI3K/Akt and PKD-NF-KB [75]. As mentioned above, a redox imbalance can also exert a toxic effect on neoplastic cells. Oxidative stress can promote senescence and cell death at different stages of cancer development. In order to counterbalance the risk of cellular toxicity mediated by the increase in ROS, cancer cells can, however, acquire the ability to upregulate antioxidant pathways and/or reprogram metabolism to increase antioxidant factors such as NADPH and reduced glutathione (GSH) [74].

Oxidative stress can act as a protumor factor at different levels, from increasing cancer risk to favoring the metastasis spreading process [74]. In fact, findings from animal models provide strong evidence about the role of ROS in increasing cancer risk due to DNA damage [76]. Mice deficient in some of the most relevant antioxidant enzymes, such as the superoxide dismutase (SOD) family (in particular the cytoplasmic SOD1 and the mitochondrial SOD2), as well as peroxiredoxin 1, developed different cancers spontaneously [77]. *Sod1*-deficient mice in particular developed liver cancer in the presence of severe oxidative and DNA damage [78].

Further evidence supporting the pivotal role of ROS in carcinogenesis derives from a countercheck test. When ROS are reduced or suppressed by drugs, for example, N-acetylcysteine (NAC), the development of cancer is attenuated in specific mouse models with premalignant genetic lesions [79,80]. However, the ROS levels should not exceed an upper limit for exerting their tumorigenic effect and not precipitating apoptosis or senescence [74].

Cogent scientific findings that ROS are both carcinogenic and cytotoxic make their modulation a promising therapeutic strategy in cancer patients. Nonetheless, the complex molecular mechanisms governing the final result are many, including their level, metabolic milieu, and altered pathways. As such, in any specific tumor, either antioxidants or prooxidants may exert antitumoral activity and be effective oncological drugs. The potential enhanced effect of chemotherapeutic drugs in the presence of antioxidants is interesting. Different compounds (nutrients, extracts from plants, enzymes) known to be relevant sources of antioxidants have been tested in order to increase the activity of anticancer drugs, both to enhance cytotoxicity and alleviate unwanted toxicity to vital tissues [81,82,83,84,85]. However, the beneficial role of antioxidants associated with chemotherapy is still debated, regarding their alleged and already mentioned protective role towards cancer cells [86]

## 7. Neurofibromin and Oxidative Stress

**Figure 4:**
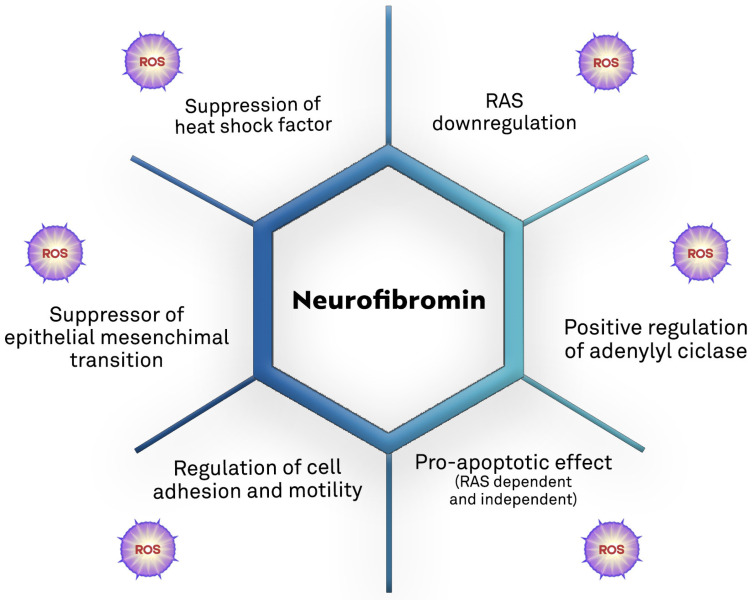
Biological mechanisms of the tumor suppression action of neurofibromin and the potential direct and/or indirect contribution of ROS [7,60,87,88,89,90,91,92].

Growing evidence supports the role of oxidative stress in *NF1*-related pathogenesis (Table 1 and Figure 4).

In the course of neoplastic progression, the so-called metabolic rewiring, an adaptive behavior that allows tumoral cells to hyperproliferate in unfavorable metabolic environments such as hypoxia and low nutrient conditions, has been proposed. A suggested role for metabolic rewiring in the development of *NF1*-related tumors has been reported, especially in the transformation of neurofibromas into MPNSTs, during which an increased avidity for glucose has been described. Such a finding supports the hypothesis of increased glycolytic processes as well as a contextual down-regulation of oxidative phosphorylation [97].

The proliferation of Ras-activated cells seems to be dependent on ROS [7]. Both neurofibromin loss and Ras activation in oligodendrocytes induced reversible ROS increases and endothelial tight junction disruption, which could be withdrawn by daily antioxidant treatment [94]. Similarly, neurofibromin-deficient macrophages and monocytic cells showed disproportionate Ras-dependent ROS production and oxidative DNA damage [95].

Loss of *NF1* has been correlated with impaired respiration and increased glycolysis, in an ERK-dependent fashion, and involving the mitochondrial chaperone tumor necrosis factor receptor-associated protein 1 (TRAP1). Such alterations resulted in the inhibition of succinate dehydrogenase (SDH) and a consequent stabilization of hypoxia-inducible factor-1 (HIF1) α levels, with the induction of a pseudohypoxic transcriptional program required for *NF1*-related tumor growth [96,98]. A particular role seems to be acted by the tumor suppressor sirtuin-3 (*SIRT3)*, which encodes for one of the most prominent deacetylases. It can regulate acetylation levels in mitochondria, playing apparent dissonant functions in cancer development by both promoting and hampering cancer cell development [97]. Among the different factors contributing to the bivalent functions of *SIRT3* in cancer, there is NAD+ availability. In a more recent study, Masgras et al. demonstrated how the loss of neurofibromin can result in an unbalanced intracellular NAD/NADPH ratio, with an important role for the increased intracellular NAD+, in addition to the reactivation of *SIRT3* and in synergy with *TRAP1* inhibition, in contrast to the proliferation of *NF1*-related neoplasms [97]. Furthermore, *SIRT3* also shows antioxidant properties, and acts as an antagonist of HIF1 alpha-induced protumoral phenotypes [97].

*NF1* loss promotes tumorigenesis by also activating heat shock factor 1 (HSF1), the master transcriptional regulator of the heat shock response [89]. In normal conditions, HSF1 is constitutively expressed in many cells and is usually maintained in its monomeric, inactive form when unstimulated. Under oxidative stress, it could be activated, with a consequent increased production of protective HS proteins [90]. *NF1*-deficient cells become tolerant to proteotoxic stress and, in animal models, *HSF1* loss of function contrasts the development of *NF1*-related cancers by weakening oncogenic Ras/MAPK signaling. Furthermore, overexpression and general activation of HSF1 have been reported in *NF1*-related MPNSTs [89]. In brief, a condition of oxidative stress could be indirectly related to the development of cancer in *NF1*-deficient cells by the increased activity of HSF1.

Studies on *Drosophila melanogaster* models have revealed further relationships between *NF1* and ROS [93]. Specifically, *NF1* overexpression in flies prolonged lifespan, enhanced reproductive health, and potentiated resistance to heat and oxidative stress by increasing mitochondrial respiration and reducing ROS levels. The adenylyl cyclase/cyclic adenosine monophosphate (cAMP)/protein kinase A pathway has been shown to directly regulate these fly phenotypes [93]. Contextually, cAMP analog drugging lengthened the lives of wild-type *D. melanogaster*, while antioxidants that catalyze metalloporphyrin oxidation restored the lifespan of *NF1* mutants [93]. Based on these data, neurofibromin regulates lifespan and stress tolerance through cAMP modulation of mitochondrial respiration and ROS generation; moreover, catalytic antioxidants may manage *NF1*-related manifestations.

Considering the signaling cascade involved in *NF1*-related cancers, interestingly, Ras activation is known to enhance ROS production in normal and neoplastic cells [99,100]. In particular, the Ras downstream kinases Akt and Erk directly stimulate superoxide and ROS production in phagocytes [101,102,103]. In addition, the mTOR pathway is involved in reducing glutathione, since mTOR specifically controls sterol regulatory element-binding proteins (SREBP), a transcription factor that manages the synthesis of glucose-6-phosphate dehydrogenase (G6PD), a rate-limiting enzyme in NADPH synthesis [8]. NADPH is indeed essential in several antioxidative systems, including the function of glutathione reductase, a crucial enzyme in maintaining intracellular redox balance and preventing oxidative damage [104].

On the other hand, ROS production may trigger apoptosis promoted by the tumor necrosis factor-related apoptosis-inducing ligand (TRAIL), a powerful tumor-specific apoptosis inducer [87,105]. Actually, the increased ROS levels boosted TRAIL-mediated apoptosis and TRAIL sensitivity in *NF1*-deficient MPNST cell lines [87]. This molecular synergy is promising for developing new therapeutic combinations.

In addition, some *NF1*-related cancers have been demonstrated to be sensitive to the toxic activity mediated by ROS. De Raedt et al. demonstrated, for example, that MPNSTs are sensitive to compounds inducing stress in the endoplasmic reticulum (ER), with a vicious circle in which the damaged ER produced ROS which, in turn, induced further ER damage [7].

Using a yeast platform to high-throughput screen synthetic lethality in yeast with *NF1* homolog (*IRA2*) deficiency revealed that one of the main selective candidates was Y100, an isoxazoloanthrone [106]. In vitro cell line studies on its action mechanisms showed that this small compound induced mitochondrial superoxide and consequent oxidative stress and DNA damage responses, resulting in cell death [106]. These data further support the key role of oxidative balance in the growth of *NF1*-deficient tumors.

Finally, the contribution of other factors, such as mutations in other tumor genes such as *TP53* or *PTEN*, as amplifiers of the *NF1*-related cancer cell ROS sensitivity, remains an unexplored research field [106].

## 8. Therapeutic Implications of *NF1*-Related Oxidative Damage

Medical treatment for *NF1* patients has been historically limited. However, the progressive improvement in knowledge of the molecular mechanisms involved in the pathogenesis of the disease has led to attempts to use targeted drugs. Blocking aberrant Ras signaling has been one of the most studied options, as has the use of drugs targeting the mTOR pathway. In 2020, the United States Food and Drug Administration (FDA) approved selumetinib, a MEK inhibitor, in patients at least 2 years old with symptomatic and inoperable plexiform neurofibromas [107,108]. Several trials with other MEK inhibitors, also for topical use, are ongoing (see Table 2). In tumor cells, MEK inhibitors activate mitochondrial oxidative metabolism by stimulating ROS production [109,110].

As regards medical therapy, a promising strategy is to target oxidative stress since it is directly or indirectly involved in many of the more relevant protumoral mechanisms implied in the development of *NF1*-related tumors. The hypothesis that antioxidants may improve some phenotypes in the so-called RASopathies has been investigated [94]. Notably, treatments that finally result in a significant increase in ROS levels could be likewise effective in promoting apoptosis.

Zhang et al. reported that lipoamide, the neutral amide of α-lipoic acid with strong scavenging activity on ROS, was able to inhibit, in a dose-dependent manner, both the *NF1*-related and the spontaneous epithelial–mesenchymal transition [92].

Pertinent findings resulted from a study performed in order to increase the sensitivity of MPNST cells to TRAIL. In particular, the authors tested curcumin, a turmeric-derived polyphenol whose effect is probably, in such a case, mediated by an increased production of ROS, with positive results on the sensitivity to TRAIL and increased apoptosis induced by TRAIL [87]. Such an improved sensitivity was also blocked by NAC, a well-known antioxidant, and conversely mimicked by exogenous ROS [87]. The latter findings represent a relevant countercheck for the hypothesized mechanism of action of curcumin.

In vitro studies have observed the killing of *NF1*-deficient nervous system cancer cells by a combination of sapanisert, a mTOR inhibitor, and vorinostat, a histone deacetylase (HDAC) inhibitor [112]. This effect seemed to be mediated by irresolvable oxidative stress, specifically derived from peculiar synergistic mechanisms, resulting in cell death and tumor regression. Indeed, the mTOR inhibitor alone was able to suppress the glutathione pathway, while its association with a HDAC inhibitor induced the expression of the thioredoxin-interacting protein (TXNIP), which is able to inhibit thioredoxin, another major antioxidant pathway in tumors. As a consequence, this combination produced a cascade of events ending in catastrophic oxidative stress.

Some results regard the potential role of compounds targeting oxidative stress in the management of *NF1*-related phenotypes, useful for promoting clinical trials in humans, were derived from animal models. Inhibitors of nitric oxide synthase seem to rescue the myelin decompaction observed in adult mice with *NF1*-deficient oligodendrocytes [113]. Based on this evidence and a suggested role for NAC in reducing nervous impairment, two trials are ongoing: a phase II to explore the safety, tolerability, and efficacy of NAC on motor behavior and/or learning in *NF1* children aged 8 through 16 years old (NCT04481048), and another to evaluate learning and motor behavior in children with *NF1* in treatment with NAC (NCT04481035).

The potential efficacy of metalloporphyrin catalytic antioxidants in the treatment of *NF1* derives from a *Drosophila melanogaster* model. Specifically, these compounds restored the lifespan of *NF1*-deleted flies [93].

The effect of an antioxidant diet was also studied. The Mediterranean diet is characterized by a significant presence of polyphenols, especially those contained in olive oil, with strong antioxidant abilities, whereas curcumin is an important phytochemical present in the Indian diet. The impact of the Mediterranean diet versus the Western diet on the *NF1* patients’ health, either with or without supplementation of curcumin, was compared in a study by Esposito et al. [111]. Despite the small number of patients (only 11), a beneficial effect of adding curcumin to the Mediterranean diet for at least six months was reported, with a significant reduction in the number and volume of cutaneous neurofibromas as compared with the other diet regimens. Currently, a new phase I study is ongoing (NCT05363267) aimed at evaluating the effect of a nutraceutical intervention with high-phenolic extra virgin olive oil and curcumin in *NF1* adult patients with cutaneous neurofibromas.

Photodynamic therapy (PDT) is a therapeutic option for specific oncological diseases, in particular cutaneous lesions [117]. Its aim is to generate cytotoxic free radicals by combining the effects of a photosensitizer compound with light stimulation at an appropriate wavelength. Although, to date, PDT has not been selectively approved as a treatment for cancer associated with *NF1*, some studies have demonstrated promising results with different sensitizers, in some cases also associated with systemic drugs [114,115,116].

The most relevant challenge of taking advantage of the oxidative vulnerability of cancer as a therapeutic option is the double-edged sword potential that characterizes drugs acting on oxidative metabolism, both pro- and antitumoral. The same compound could, for example, act differently at different stages of the natural history of cancer development and spreading, depending on its association with other molecules and the specific desired target. Ideally, the main goal of future research should be to better understand how and when a supposed “switch” is able to drive in the desired direction. 

## 9. Conclusions

Many signaling pathways are involved in the molecular pathogenesis of *NF1*-altered tumors, both sporadic and inherited, associated with the genodermatosis *NF1*.

Nowadays, when a specific oncogene mutation is related to cancer development, molecular target therapy seems like the best option for its treatment. Nevertheless, tumor suppressor genes, such as *NF1* and *TP53*, favor tumorigenesis with their loss and are, therefore, difficult to treat with direct enzyme inhibitors or antibodies. Recently, roundabout therapeutic strategies have turned out to be successful, such as PARP inhibitors in *BRCA1-* and *BRCA2-*mutated tumors or immunotherapy in tumors with mismatch mutations. A single mutation elicits a very large number of downstream events, each of which is potentially affected or targetable by a variety of reactions and molecules.

Oxidative stress is the result of an imbalance between reductive and oxidative reactions, which ends with the production of ROS with opposite consequences. ROS may exert an important cytotoxic action, which can promote degeneration and carcinogenesis or otherwise induce apoptosis. In different diseases, such as cardiovascular or neurodegenerative conditions, the importance of oxidative stress and ROS has been well studied, not only for a better comprehension of their pathogenesis, but especially to develop new therapeutic strategies.

In *NF1*-altered tumors, the role of oxidative stress and ROS in both the pathogenesis and targetable vulnerability is emerging. Among the mechanisms by which the loss of the oncosuppressor neurofibromin exerts its protumoral effects are oxidative stress and elevated ROS. Unfortunately, studies aiming to determine the therapeutical advantages of ROS are not easy to design, mostly due to their two-faced Janus properties, both tumorigenic and antitumoral, depending on their level, metabolism milieu, and altered pathways. In this field, nutraceutical interventions are a promising option for preventing or delaying cancer. Therefore, further studies are needed in order to better clarify, and maybe selectively potentiate, the cytotoxic and antitumoral effects of ROS intervention as well as the protective role of antioxidant compounds in preventing and contrasting the development of *NF1*-related cancers.

## Figures and Tables

**Table 1:** Primary modifications of the oxidative metabolism observed in *NF1*-deficient models.

Cell Type	Oxidative Effect	Molecular Mechanisms	Reference
*Drosophila melanogaster*	↑ ROS	↑ Adenylyl cyclase ↑ cAMP	[93]
MPNST cell lines	↑ ER stress levels	↑ Ras pathway ↑ mTOR pathway	[7]
MEFs MPNST cell lines	Proteotoxic stress tolerance	↑ Ras/MAPK pathway ↑ HSF1	[89]
Mouse oligodendrocytes	↑ ROS	↑Ras pathway ↑NO synthase	[94]
Mouse macrophages Human monocytic cells	↑ ROS	↑Ras pathway	[95]
MEFs U87 glioblastoma cell line	↑ Glycolysis	↓ SDH ↑ ERK-dependent TRAP1 ↑ HIF1α	[96]
MEFs MPNST cell lines	↑ ROS	↓ ERK-dependent NADPH dehydrogenase ↓ NAD+-dependent SIRT3 ↑ ERK-dependent TRAP1	[97]

Acronyms: ROS, reactive oxygen species; cAMP, cyclic adenosine monophosphate; MPNST, malignant peripheral nerve sheath tumor; ER, endoplasmic reticulum; mTOR, mechanistic target of rapamycin; MEFs, mouse embryonic fibroblasts; MAPK, mitogen-activated protein kinase; HSF1, heat shock factor 1; NO, nitric oxide; SDH, succinate dehydrogenase; TRAP1, tumor necrosis factor receptor associated protein 1; HIF1α, hypoxia-inducible factor-1α; NAPDH, nicotinamide adenine dinucleotide phosphate; SIRT3, sirtuin-3.

**Table 2:** The principal studied compounds modulating oxidative stress in *NF1*-deficient conditions.

Compounds	Mechanism of Action	Setting	Reference
Curcumin	Oxidative stress	MPSNT cell lines	[87]
Lipoamide	Antioxidant	Mouse Schwann cells Mouse model	[92]
Mediterranean diet and curcumin	Antioxidant	Clinical study (case series, cutaneous neurofibromas)	[111]
Metalloporphyrin catalytic	Antioxidant	*Drosophila melanogaster* model	[93]
mTOR inhibitor + HDAC inhibitor	Catastrophic oxidative stress	MPNST cell lines NSCLC cell line Mouse model	[112]
NOS inhibitors	Antioxidant	Mouse model	[113]
PDT with ALA + doxycycline	Oxidative stress	MPNST cell line	[114]
PDT + cisplatin	Oxidative stress	MPNST cell line MPNST mouse xenograft	[115]
PDT with ALA	Oxidative stress	Phase I (cutaneous neurofibromas)	[116]
PI-504	HSP90inhibitors	MPNST cell lines	[7]
Selumetinib	MEK inhibitor	FDA approved for plexiform neurofibromas	[108]
Thapsigargin	ER calcium-ATPase inhibitor	MPNST cell lines	[7]
TRAIL	ROS-influenced Proapoptotic	MPNST cell lines	[87]
Tunicamycin	Glycosylation inhibitor	MPNST cell lines	[7]
Y100	Oxidative stress		[106]
Ongoing clinical trials			
Binimetinib	MEK inhibitor	Phase 1 (plexiform neurofibromas)	NCT03231306
FCN-159	MEK inhibitor	Phase 3 (inoperable plexiform neurofibromas)	NCT05913037
Mediterranean diet with curcumin	Antioxidant	Phase 1 (*NF1*-related neurofibromas)	NCT05363267
Mirdametinib	MEK inhibitor	Phase 2b (inoperable plexiform neurofibromas)	NCT03962543
NAC	Antioxidant	Phase 2a (learning and motor behavior in children with *NF1*) Phase 2 (learning and motor behavior in children with *NF1*)	NCT04481035 NCT04481048
NFX-179	Topical MEK inhibitor	Phase 2a (*NF1*-related cutaneous neurofibromas	NCT04435665 NCT05005845
TQ-B3234	MEK inhibitor	Phase 1 (*NF1*-related neurofibromas and MPSNT)	NCT05107037
Trametinib	MEK inhibitor	Single arm, open-label plexiform neurofibromas)	NCT03741101

Acronyms: MPNST, malignant peripheral nerve sheath tumor; mTOR, mechanistic target of rapamycin; HDAC, histone-deacetylase; NSCLC, non-small cell lung cancer; NOS, nitric oxide synthase; PDT, Photodynamic Therapy; ALA, aminolevulinic acid; HSP90, heat shock protein 90; ER, endoplasmic reticulum; FDA, United States Food and Drug Administration; ATP, adenosine triphosphate; TRAIL, tumor necrosis factor-related apoptosis inducing ligand; ROS, reactive oxygen species; *NF1*, neurofibromatosis type 1; NAC, N-acetyl-cysteine.

## References

[1] Sabbagh A., Pasmant E., Imbard A., Luscan A., Soares M., Blanché H., Laurendeau I., Ferkal S., Vidaud M., Pinson S. (2013). *NF1* molecular characterization and neurofibromatosis type I genotype-phenotype correlation: The French experience. Hum. Mutat..

[2] Trachootham D., Lu W., Ogasawara M.A., Nilsa R.D., Huang P. (2008). Redox regulation of cell survival. Antioxid. Redox. Signal..

[3] Gorrini C., Harris I.S., Mak T.W. (2013). Modulation of oxidative stress as an anticancer strategy. Nat. Rev. Drug Discov..

[4] Thanan R., Oikawa S., Hiraku Y., Ohnishi S., Ma N., Pinlaor S., Yongvanit P., Kawanishi S., Murata M. (2014). Oxidative stress and its significant roles in neurodegenerative diseases and cancer. Int. J. Mol. Sci..

[5] Ohba Y., Mochizuki N., Yamashita S., Chan A.M., Schrader J.W., Hattori S., Nagashima K., Matsuda M. (2000). Regulatory proteins of R-Ras, TC21/R-Ras2, and M-Ras/R-Ras3. J. Biol. Chem..

[6] Li X., Gao M., Choi J.M., Kim B.J., Zhou M.T., Chen Z., Jain A.N., Jung S.Y., Yuan J., Wang W. (2017). Clustered, Regularly Interspaced Short Palindromic Repeats (CRISPR)/Cas9-coupled Affinity Purification/Mass Spectrometry Analysis Revealed a Novel Role of Neurofibromin in mTOR Signaling. Mol. Cell. Proteom..

[7] De Raedt T., Walton Z., Yecies J.L., Li D., Chen Y., Malone C.F., Maertens O., Jeong S.M., Bronson R.T., Lebleu V. (2011). Exploiting cancer cell vulnerabilities to develop a combination therapy for ras-driven tumors. Cancer Cell.

[8] Düvel K., Yecies J.L., Menon S., Raman P., Lipovsky A.I., Souza A.L., Triantafellow E., Ma Q., Gorski R., Cleaver S. (2010). Activation of a metabolic gene regulatory network downstream of mTOR complex 1. Mol. Cell.

[9] Brosius S. (2010). A history of von Recklinghausen’s *NF1*. J. Hist. Neurosci..

[10] Easton D.F., Ponder M.A., Huson S.M., Ponder B.A. (1993). An analysis of variation in expression of neurofibromatosis (NF) type 1 (*NF1*): Evidence for modifying genes. Am. J. Hum. Genet..

[11] DeBella K., Szudek J., Friedman J.M. (2000). Use of the national institutes of health criteria for diagnosis of neurofibromatosis 1 in children. Pediatrics.

[12] Friedman J.M., Arbiser J., Epstein J.A., Gutmann D.H., Huot S.J., Lin A.E., McManus B., Korf B.R. (2002). Cardiovascular disease in neurofibromatosis 1: Report of the *NF1* Cardiovascular Task Force. Genet. Med..

[13] Hyman S.L., Arthur Shores E., North K.N. (2006). Learning disabilities in children with neurofibromatosis type 1: Subtypes, cognitive profile, and attention-deficit-hyperactivity disorder. Dev. Med. Child Neurol..

[14] Seminog O.O., Goldacre M.J. (2013). Risk of benign tumours of nervous system, and of malignant neoplasms, in people with neurofibromatosis: Population-based record-linkage study. Br. J. Cancer.

[15] Rasmussen S.A., Yang Q., Friedman J.M. (2001). Mortality in neurofibromatosis 1: An analysis using U.S. death certificates. Am. J. Hum. Genet..

[16] Landry J.P., Schertz K.L., Chiang Y.J., Bhalla A.D., Yi M., Keung E.Z., Scally C.P., Feig B.W., Hunt K.K., Roland C.L. (2021). Comparison of Cancer Prevalence in Patients with Neurofibromatosis Type 1 at an Academic Cancer Center vs in the General Population from 1985 to 2020. JAMA Netw. Open.

[17] Stiller C.A., Chessells J.M., Fitchett M. (1994). Neurofibromatosis and childhood leukaemia/lymphoma: A population-based UKCCSG study. Br. J. Cancer.

[18] Carton C., Evans D.G., Blanco I., Friedrich R.E., Ferner R.E., Farschtschi S., Salvador H., Azizi A.A., Mautner V., Röhl C. (2023). ERN GENTURIS tumour surveillance guidelines for individuals with neurofibromatosis type 1. EClinicalMedicine.

[19] Pasmant E., Vidaud M., Vidaud D., Wolkenstein P. (2012). Neurofibromatosis type 1: From genotype to phenotype. J. Med. Genet..

[20] Upadhyaya M., Ruggieri M., Maynard J., Osborn M., Hartog C., Mudd S., Penttinen M., Cordeiro I., Ponder M., Ponder B.A. (1998). Gross deletions of the neurofibromatosis type 1 (*NF1*) gene are predominantly of maternal origin and commonly associated with a learning disability, dysmorphic features and developmental delay. Hum. Genet..

[21] Koczkowska M., Chen Y., Callens T., Gomes A., Sharp A., Johnson S., Hsiao M.C., Chen Z., Balasubramanian M., Barnett C.P. (2018). Genotype-Phenotype Correlation in *NF1*: Evidence for a More Severe Phenotype Associated with Missense Mutations Affecting *NF1* Codons 844-848. Am. J. Hum. Genet..

[22] Upadhyaya M., Huson S.M., Davies M., Thomas N., Chuzhanova N., Giovannini S., Evans D.G., Howard E., Kerr B., Griffiths S. (2007). An absence of cutaneous neurofibromas associated with a 3-bp inframe deletion in exon 17 of the *NF1* gene (c.2970-2972 delAAT): Evidence of a clinically significant *NF1* genotype-phenotype correlation. Am. J. Hum. Genet..

[23] Pinna V., Lanari V., Daniele P., Consoli F., Agolini E., Margiotti K., Bottillo I., Torrente I., Bruselles A., Fusilli C. (2015). p. Arg1809Cys substitution in neurofibromin is associated with a distinctive *NF1* phenotype without neurofibromas. Eur. J. Hum. Genet..

[24] Rojnueangnit K., Xie J., Gomes A., Sharp A., Callens T., Chen Y., Liu Y., Cochran M., Abbott M.A., Atkin J. (2015). High Incidence of Noonan Syndrome Features Including Short Stature and Pulmonic Stenosis in Patients carrying *NF1* Missense Mutations Affecting p.Arg1809: Genotype-Phenotype Correlation. Hum. Mutat..

[25] Koczkowska M., Callens T., Chen Y., Gomes A., Hicks A.D., Sharp A., Johns E., Uhas K.A., Armstrong L., Bosanko K.A. (2020). Clinical spectrum of individuals with pathogenic *NF1* missense variants affecting p.Met1149, p.Arg1276, and p.Lys1423: Genotype-phenotype study in neurofibromatosis type 1. Hum. Mutat..

[26] Side L., Taylor B., Cayouette M., Conner E., Thompson P., Luce M., Shannon K. (1997). Homozygous inactivation of the *NF1* gene in bone marrow cells from children with neurofibromatosis type 1 and malignant myeloid disorders. N. Engl. J. Med..

[27] Sawada S., Florell S., Purandare S.M., Ota M., Stephens K., Viskochil D. (1996). Identification of *NF1* mutations in both alleles of a dermal neurofibroma. Nat. Genet..

[28] Vogelstein B., Papadopoulos N., Velculescu V.E., Zhou S., Diaz L.A., Kinzler K.W. (2013). Cancer genome landscapes. Science.

[29] Cerami E., Gao J., Dogrusoz U., Gross B.E., Sumer S.O., Aksoy B.A., Jacobsen A., Byrne C.J., Heuer M.L., Larsson E. (2012). The cBio cancer genomics portal: An open platform for exploring multidimensional cancer genomics data. Cancer Discov..

[30] Martelotto L.G., De Filippo M.R., Ng C.K., Natrajan R., Fuhrmann L., Cyrta J., Piscuoglio S., Wen H.C., Lim R.S., Shen R. (2015). Genomic landscape of adenoid cystic carcinoma of the breast. J. Pathol..

[31] Lee W., Teckie S., Wiesner T., Ran L., Prieto Granada C.N., Lin M., Zhu S., Cao Z., Liang Y., Sboner A. (2014). PRC2 is recurrently inactivated through EED or SUZ12 loss in malignant peripheral nerve sheath tumors. Nat. Genet..

[32] Network C.G.A. (2015). Genomic Classification of Cutaneous Melanoma. Cell.

[33] Shain A.H., Garrido M., Botton T., Talevich E., Yeh I., Sanborn J.Z., Chung J., Wang N.J., Kakavand H., Mann G.J. (2015). Exome sequencing of desmoplastic melanoma identifies recurrent NFKBIE promoter mutations and diverse activating mutations in the MAPK pathway. Nat. Genet..

[34] Chang D., Shain A.H. (2021). The landscape of driver mutations in cutaneous squamous cell carcinoma. NPJ Genom. Med..

[35] Gambini D., Passoni E., Nazzaro G., Beltramini G., Tomasello G., Ghidini M., Kuhn E., Garrone O. (2022). Basal Cell Carcinoma and Hedgehog Pathway Inhibitors: Focus on Immune Response. Front. Med..

[36] Bonilla X., Parmentier L., King B., Bezrukov F., Kaya G., Zoete V., Seplyarskiy V.B., Sharpe H.J., McKee T., Letourneau A. (2016). Genomic analysis identifies new drivers and progression pathways in skin basal cell carcinoma. Nat. Genet..

[37] Wang L.B., Karpova A., Gritsenko M.A., Kyle J.E., Cao S., Li Y., Rykunov D., Colaprico A., Rothstein J.H., Hong R. (2021). Proteogenomic and metabolomic characterization of human glioblastoma. Cancer Cell.

[38] Verhaak R.G., Hoadley K.A., Purdom E., Wang V., Qi Y., Wilkerson M.D., Miller C.R., Ding L., Golub T., Mesirov J.P. (2010). Integrated genomic analysis identifies clinically relevant subtypes of glioblastoma characterized by abnormalities in PDGFRA, IDH1, EGFR, and *NF1*. Cancer Cell.

[39] Kuhn E., Kurman R.J., Vang R., Sehdev A.S., Han G., Soslow R., Wang T.L., Shih Ie M. (2012). TP53 mutations in serous tubal intraepithelial carcinoma and concurrent pelvic high-grade serous carcinoma—Evidence supporting the clonal relationship of the two lesions. J. Pathol..

[40] Network C.G.A.R. (2011). Integrated genomic analyses of ovarian carcinoma. Nature.

[41] Hoadley K.A., Yau C., Hinoue T., Wolf D.M., Lazar A.J., Drill E., Shen R., Taylor A.M., Cherniack A.D., Thorsson V. (2018). Cell-of-Origin Patterns Dominate the Molecular Classification of 10,000 Tumors from 33 Types of Cancer. Cell.

[42] Trovó-Marqui A.B., Tajara E.H. (2006). Neurofibromin: A general outlook. Clin. Genet..

[43] Wimmer K., Yao S., Claes K., Kehrer-Sawatzki H., Tinschert S., De Raedt T., Legius E., Callens T., Beiglböck H., Maertens O. (2006). Spectrum of single- and multiexon *NF1* copy number changes in a cohort of 1,100 unselected *NF1* patients. Genes Chromosomes Cancer.

[44] Martín Y., Dopazo A., Hernández-Chico C. (2011). Progress and challenges in developing a molecular diagnostic test for neurofibromatosis type 1. Expert. Rev. Mol. Diagn..

[45] Luijten M., Wang Y., Smith B.T., Westerveld A., Smink L.J., Dunham I., Roe B.A., Hulsebos T.J. (2000). Mechanism of spreading of the highly related neurofibromatosis type 1 (*NF1*) pseudogenes on chromosomes 2, 14 and 22. Eur. J. Hum. Genet..

[46] Ars E., Serra E., García J., Kruyer H., Gaona A., Lázaro C., Estivill X. (2000). Mutations affecting mRNA splicing are the most common molecular defects in patients with neurofibromatosis type 1. Hum. Mol. Genet..

[47] Messiaen L.M., Callens T., Mortier G., Beysen D., Vandenbroucke I., Van Roy N., Speleman F., Paepe A.D. (2000). Exhaustive mutation analysis of the *NF1* gene allows identification of 95% of mutations and reveals a high frequency of unusual splicing defects. Hum. Mutat..

[48] Peltonen J., Jaakkola S., Lebwohl M., Renvall S., Risteli L., Virtanen I., Uitto J. (1988). Cellular differentiation and expression of matrix genes in type 1 neurofibromatosis. Lab. Investig..

[49] Koczkowska M., Chen Y., Xie J., Callens T., Gomes A., Wimmer K., Messiaen L.M. (2023). Analysis of 200 unrelated individuals with a constitutional *NF1* deep intronic pathogenic variant reveals that variants flanking the alternatively spliced *NF1* exon 31 [23a] cause a classical neurofibromatosis type 1 phenotype while altering predominantly *NF1* isoform type II. Hum. Genet..

[50] Kluwe L., Siebert R., Gesk S., Friedrich R.E., Tinschert S., Kehrer-Sawatzki H., Mautner V.F. (2004). Screening 500 unselected neurofibromatosis 1 patients for deletions of the *NF1* gene. Hum. Mutat..

[51] Jacks T., Shih T.S., Schmitt E.M., Bronson R.T., Bernards A., Weinberg R.A. (1994). Tumour predisposition in mice heterozygous for a targeted mutation in *Nf1*. Nat. Genet..

[52] Brannan C.I., Perkins A.S., Vogel K.S., Ratner N., Nordlund M.L., Reid S.W., Buchberg A.M., Jenkins N.A., Parada L.F., Copeland N.G. (1994). Targeted disruption of the neurofibromatosis type-1 gene leads to developmental abnormalities in heart and various neural crest-derived tissues. Genes Dev..

[53] Tong S., Devine W.P., Shieh J.T. (2022). Tumor and Constitutional Sequencing for Neurofibromatosis Type 1. JCO Precis. Oncol..

[54] Serra E., Rosenbaum T., Nadal M., Winner U., Ars E., Estivill X., Lázaro C. (2001). Mitotic recombination effects homozygosity for *NF1* germline mutations in neurofibromas. Nat. Genet..

[55] McGillicuddy L.T., Fromm J.A., Hollstein P.E., Kubek S., Beroukhim R., De Raedt T., Johnson B.W., Williams S.M., Nghiemphu P., Liau L.M. (2009). Proteasomal and genetic inactivation of the *NF1* tumor suppressor in gliomagenesis. Cancer Cell.

[56] Khosravi T., Oladnabi M. (2023). The role of miRNAs and lncRNAs in neurofibromatosis type 1. J. Cell Biochem..

[57] Fishbein L., Eady B., Sanek N., Muir D., Wallace M.R. (2005). Analysis of somatic *NF1* promoter methylation in plexiform neurofibromas and Schwann cells. Cancer Genet. Cytogenet..

[58] Peta C., Tsirimonaki E., Samouil D., Georgiadou K., Mangoura D. (2020). Nuclear Isoforms of Neurofibromin Are Required for Proper Spindle Organization and Chromosome Segregation. Cells.

[59] Xu G.F., O’Connell P., Viskochil D., Cawthon R., Robertson M., Culver M., Dunn D., Stevens J., Gesteland R., White R. (1990). The neurofibromatosis type 1 gene encodes a protein related to GAP. Cell.

[60] Tong J., Hannan F., Zhu Y., Bernards A., Zhong Y. (2002). Neurofibromin regulates G protein-stimulated adenylyl cyclase activity. Nat. Neurosci..

[61] Nussinov R., Jang H., Zhang M., Tsai C.J., Sablina A.A. (2020). The Mystery of Rap1 Suppression of Oncogenic Ras. Trends Cancer.

[62] Dasgupta B., Yi Y., Chen D.Y., Weber J.D., Gutmann D.H. (2005). Proteomic analysis reveals hyperactivation of the mammalian target of rapamycin pathway in neurofibromatosis 1-associated human and mouse brain tumors. Cancer Res..

[63] Boyanapalli M., Lahoud O.B., Messiaen L., Kim B., Anderle de Sylor M.S., Duckett S.J., Somara S., Mikol D.D. (2006). Neurofibromin binds to caveolin-1 and regulates ras, FAK, and Akt. Biochem. Biophys. Res. Commun..

[64] Starinsky-Elbaz S., Faigenbloom L., Friedman E., Stein R., Kloog Y. (2009). The pre-GAP-related domain of neurofibromin regulates cell migration through the LIM kinase/cofilin pathway. Mol. Cell. Neurosci..

[65] Ozawa T., Araki N., Yunoue S., Tokuo H., Feng L., Patrakitkomjorn S., Hara T., Ichikawa Y., Matsumoto K., Fujii K. (2005). The neurofibromatosis type 1 gene product neurofibromin enhances cell motility by regulating actin filament dynamics via the Rho-ROCK-LIMK2-cofilin pathway. J. Biol. Chem..

[66] Kweh F., Zheng M., Kurenova E., Wallace M., Golubovskaya V., Cance W.G. (2009). Neurofibromin physically interacts with the N-terminal domain of focal adhesion kinase. Mol. Carcinog..

[67] Errico A., Stocco A., Riccardi V.M., Gambalunga A., Bassetto F., Grigatti M., Ferlosio A., Tadini G., Garozzo D., Ferraresi S. (2021). Neurofibromin Deficiency and Extracellular Matrix Cooperate to Increase Transforming Potential through FAK-Dependent Signaling. Cancers.

[68] Arima Y., Hayashi H., Kamata K., Goto T.M., Sasaki M., Kuramochi A., Saya H. (2010). Decreased expression of neurofibromin contributes to epithelial-mesenchymal transition in neurofibromatosis type 1. Exp. Dermatol..

[69] Luo G., Kim J., Song K. (2014). The C-terminal domains of human neurofibromin and its budding yeast homologs Ira1 and Ira2 regulate the metaphase to anaphase transition. Cell Cycle.

[70] Koliou X., Fedonidis C., Kalpachidou T., Mangoura D. (2016). Nuclear import mechanism of neurofibromin for localization on the spindle and function in chromosome congression. J. Neurochem..

[71] Patrakitkomjorn S., Kobayashi D., Morikawa T., Wilson M.M., Tsubota N., Irie A., Ozawa T., Aoki M., Arimura N., Kaibuchi K. (2008). Neurofibromatosis type 1 (*NF1*) tumor suppressor, neurofibromin, regulates the neuronal differentiation of PC12 cells via its associating protein, CRMP-2. J. Biol. Chem..

[72] Moutal A., Villa L.S., Yeon S.K., Householder K.T., Park K.D., Sirianni R.W., Khanna R. (2018). CRMP2 Phosphorylation Drives Glioblastoma Cell Proliferation. Mol. Neurobiol..

[73] Sies H. (2015). Oxidative stress: A concept in redox biology and medicine. Redox. Biol..

[74] Hayes J.D., Dinkova-Kostova A.T., Tew K.D. (2020). Oxidative Stress in Cancer. Cancer Cell.

[75] Moloney J.N., Cotter T.G. (2018). ROS signalling in the biology of cancer. Semin. Cell Dev. Biol..

[76] Rose Li Y., Halliwill K.D., Adams C.J., Iyer V., Riva L., Mamunur R., Jen K.Y., Del Rosario R., Fredlund E., Hirst G. (2020). Mutational signatures in tumours induced by high and low energy radiation in Trp53 deficient mice. Nat. Commun..

[77] Gill J.G., Piskounova E., Morrison S.J. (2016). Cancer, Oxidative Stress, and Metastasis. Cold Spring Harb. Symp. Quant. Biol..

[78] Elchuri S., Oberley T.D., Qi W., Eisenstein R.S., Jackson Roberts L., Van Remmen H., Epstein C.J., Huang T.T. (2005). CuZnSOD deficiency leads to persistent and widespread oxidative damage and hepatocarcinogenesis later in life. Oncogene.

[79] Song N.Y., Zhu F., Wang Z., Willette-Brown J., Xi S., Sun Z., Su L., Wu X., Ma B., Nussinov R. (2018). IKKα inactivation promotes Kras-initiated lung adenocarcinoma development through disrupting major redox regulatory pathways. Proc. Natl. Acad. Sci. USA.

[80] Bagati A., Moparthy S., Fink E.E., Bianchi-Smiraglia A., Yun D.H., Kolesnikova M., Udartseva O.O., Wolff D.W., Roll M.V., Lipchick B.C. (2019). KLF9-dependent ROS regulate melanoma progression in stage-specific manner. Oncogene.

[81] Campagna R., Pozzi V., Giorgini S., Morichetti D., Goteri G., Sartini D., Serritelli E.N., Emanuelli M. (2023). Paraoxonase-2 is upregulated in triple negative breast cancer and contributes to tumor progression and chemoresistance. Hum. Cell..

[82] Bacchetti T., Campagna R., Sartini D., Cecati M., Morresi C., Bellachioma L., Martinelli E., Rocchetti G., Lucini L., Ferretti G. (2022). *C. spinosa* L. subsp. rupestris Phytochemical Profile and Effect on Oxidative Stress in Normal and Cancer Cells. Molecules.

[83] Campagna R., Belloni A., Pozzi V., Salvucci A., Notarstefano V., Togni L., Mascitti M., Sartini D., Giorgini E., Salvolini E. (2022). Role Played by Paraoxonase-2 Enzyme in Cell Viability, Proliferation and Sensitivity to Chemotherapy of Oral Squamous Cell Carcinoma Cell Lines. Int. J. Mol. Sci..

[84] Hamza A.A., Heeba G.H., Hassanin S.O., Elwy H.M., Bekhit A.A., Amin A. (2023). Hibiscus-cisplatin combination treatment decreases liver toxicity in rats while increasing toxicity in lung cancer cells via oxidative stress- apoptosis pathway. Biomed. Pharmacother..

[85] Tchounwou P.B., Dasari S., Noubissi F.K., Ray P., Kumar S. (2021). Advances in Our Understanding of the Molecular Mechanisms of Action of Cisplatin in Cancer Therapy. J. Exp. Pharmacol..

[86] Ambrosone C.B., Zirpoli G.R., Hutson A.D., McCann W.E., McCann S.E., Barlow W.E., Kelly K.M., Cannioto R., Sucheston-Campbell L.E., Hershman D.L. (2020). Dietary Supplement Use During Chemotherapy and Survival Outcomes of Patients with Breast Cancer Enrolled in a Cooperative Group Clinical Trial (SWOG S0221). J. Clin. Oncol..

[87] Reuss D.E., Mucha J., Hagenlocher C., Ehemann V., Kluwe L., Mautner V., von Deimling A. (2013). Sensitivity of malignant peripheral nerve sheath tumor cells to TRAIL is augmented by loss of *NF1* through modulation of MYC/MAD and is potentiated by curcumin through induction of ROS. PLoS ONE.

[88] Alexandrova A.Y., Kopnin P.B., Vasiliev J.M., Kopnin B.P. (2006). ROS up-regulation mediates Ras-induced changes of cell morphology and motility. Exp. Cell Res..

[89] Dai C., Santagata S., Tang Z., Shi J., Cao J., Kwon H., Bronson R.T., Whitesell L., Lindquist S. (2012). Loss of tumor suppressor *NF1* activates HSF1 to promote carcinogenesis. J. Clin. Investig..

[90] Szyller J., Bil-Lula I. (2021). Heat Shock Proteins in Oxidative Stress and Ischemia/Reperfusion Injury and Benefits from Physical Exercises: A Review to the Current Knowledge. Oxid. Med. Cell. Longev..

[91] Yap Y.S., McPherson J.R., Ong C.K., Rozen S.G., Teh B.T., Lee A.S., Callen D.F. (2014). The *NF1* gene revisited-from bench to bedside. Oncotarget.

[92] Zhang Y., Zhou R., Qu Y., Shu M., Guo S., Bai Z. (2017). Lipoamide Inhibits *NF1* Deficiency-induced Epithelial-Mesenchymal Transition in Murine Schwann Cells. Arch. Med. Res..

[93] Tong J.J., Schriner S.E., McCleary D., Day B.J., Wallace D.C. (2007). Life extension through neurofibromin mitochondrial regulation and antioxidant therapy for neurofibromatosis-1 in Drosophila melanogaster. Nat. Genet..

[94] Mayes D.A., Rizvi T.A., Titus-Mitchell H., Oberst R., Ciraolo G.M., Vorhees C.V., Robinson A.P., Miller S.D., Cancelas J.A., Stemmer-Rachamimov A.O. (2013). *Nf1* loss and Ras hyperactivation in oligodendrocytes induce NOS-driven defects in myelin and vasculature. Cell Rep..

[95] Bessler W.K., Hudson F.Z., Zhang H., Harris V., Wang Y., Mund J.A., Downing B., Ingram D.A., Case J., Fulton D.J. (2016). Neurofibromin is a novel regulator of Ras-induced reactive oxygen species production in mice and humans. Free Radic. Biol. Med..

[96] Masgras I., Ciscato F., Brunati A.M., Tibaldi E., Indraccolo S., Curtarello M., Chiara F., Cannino G., Papaleo E., Lambrughi M. (2017). Absence of Neurofibromin Induces an Oncogenic Metabolic Switch via Mitochondrial ERK-Mediated Phosphorylation of the Chaperone TRAP1. Cell Rep..

[97] Masgras I., Cannino G., Ciscato F., Sanchez-Martin C., Darvishi F.B., Scantamburlo F., Pizzi M., Menga A., Fregona D., Castegna A. (2022). Tumor growth of neurofibromin-deficient cells is driven by decreased respiration and hampered by NAD. Cell Death Differ..

[98] Sciacovelli M., Guzzo G., Morello V., Frezza C., Zheng L., Nannini N., Calabrese F., Laudiero G., Esposito F., Landriscina M. (2013). The mitochondrial chaperone TRAP1 promotes neoplastic growth by inhibiting succinate dehydrogenase. Cell Metab..

[99] Ferro E., Goitre L., Baldini E., Retta S.F., Trabalzini L. (2014). Ras GTPases are both regulators and effectors of redox agents. Methods Mol. Biol..

[100] Adachi Y., Shibai Y., Mitsushita J., Shang W.H., Hirose K., Kamata T. (2008). Oncogenic Ras upregulates NADPH oxidase 1 gene expression through MEK-ERK-dependent phosphorylation of GATA-6. Oncogene.

[101] Hoyal C.R., Gutierrez A., Young B.M., Catz S.D., Lin J.H., Tsichlis P.N., Babior B.M. (2003). Modulation of p47PHOX activity by site-specific phosphorylation: Akt-dependent activation of the NADPH oxidase. Proc. Natl. Acad. Sci. USA.

[102] El-Benna J., Dang P.M., Gougerot-Pocidalo M.A., Marie J.C., Braut-Boucher F. (2009). p47phox, the phagocyte NADPH oxidase/NOX2 organizer: Structure, phosphorylation and implication in diseases. Exp. Mol. Med..

[103] Li X.J., Goodwin C.B., Nabinger S.C., Richine B.M., Yang Z., Hanenberg H., Ohnishi H., Matozaki T., Feng G.S., Chan R.J. (2015). Protein-tyrosine phosphatase Shp2 positively regulates macrophage oxidative burst. J. Biol. Chem..

[104] Ju H.Q., Lin J.F., Tian T., Xie D., Xu R.H. (2020). NADPH homeostasis in cancer: Functions, mechanisms and therapeutic implications. Signal. Transduct. Target Ther..

[105] Mellier G., Pervaiz S. (2012). The three Rs along the TRAIL: Resistance, re-sensitization and reactive oxygen species (ROS). Free Radic Res..

[106] Allaway R.J., Wood M.D., Downey S.L., Bouley S.J., Traphagen N.A., Wells J.D., Batra J., Melancon S.N., Ringelberg C., Seibel W. (2018). Exploiting mitochondrial and metabolic homeostasis as a vulnerability in *NF1* deficient cells. Oncotarget.

[107] Ciombor K.K., Bekaii-Saab T. (2015). Selumetinib for the treatment of cancer. Expert Opin. Investig. Drugs.

[108] Casey D., Demko S., Sinha A., Mishra-Kalyani P.S., Shen Y.L., Khasar S., Goheer M.A., Helms W.S., Pan L., Xu Y. (2021). FDA Approval Summary: Selumetinib for Plexiform Neurofibroma. Clin. Cancer Res..

[109] Decroocq J., Birsen R., Montersino C., Chaskar P., Mano J., Poulain L., Friedrich C., Alary A.S., Guermouche H., Sahal A. (2022). RAS activation induces synthetic lethality of MEK inhibition with mitochondrial oxidative metabolism in acute myeloid leukemia. Leukemia.

[110] Feng J., Lian Z., Xia X., Lu Y., Hu K., Zhang Y., Liu Y., Hu L., Yuan K., Sun Z. (2023). Targeting metabolic vulnerability in mitochondria conquers MEK inhibitor resistance in *KRAS*-mutant lung cancer. Acta Pharm. Sin. B.

[111] Esposito T., Schettino C., Polverino P., Allocca S., Adelfi L., D’Amico A., Capaldo G., Varriale B., Di Salle A., Peluso G. (2017). Synergistic Interplay between Curcumin and Polyphenol-Rich Foods in the Mediterranean Diet: Therapeutic Prospects for Neurofibromatosis 1 Patients. Nutrients.

[112] Malone C.F., Emerson C., Ingraham R., Barbosa W., Guerra S., Yoon H., Liu L.L., Michor F., Haigis M., Macleod K.F. (2017). mTOR and HDAC Inhibitors Converge on the TXNIP/Thioredoxin Pathway to Cause Catastrophic Oxidative Stress and Regression of RAS-Driven Tumors. Cancer Discov..

[113] Asleh J., Shofty B., Cohen N., Kavushansky A., López-Juárez A., Constantini S., Ratner N., Kahn I. (2020). Brain-wide structural and functional disruption in mice with oligodendrocyte-specific. Proc. Natl. Acad. Sci. USA.

[114] Lee M.J., Hung S.H., Huang M.C., Tsai T., Chen C.T. (2017). Doxycycline potentiates antitumor effect of 5-aminolevulinic acid-mediated photodynamic therapy in malignant peripheral nerve sheath tumor cells. PLoS ONE.

[115] Chen C.T., Peng P.C., Tsai T., Chien H.F., Lee M.J. (2020). A Novel Treatment Modality for Malignant Peripheral Nerve Sheath Tumor Using a Dual-Effect Liposome to Combine Photodynamic Therapy and Chemotherapy. Pharmaceutics.

[116] Quirk B., Olasz E., Kumar S., Basel D., Whelan H. (2021). Photodynamic Therapy for Benign Cutaneous Neurofibromas Using Aminolevulinic Acid Topical Application and 633 nm Red Light Illumination. Photobiomodul. Photomed. Laser Surg..

[117] Allison R.R., Moghissi K. (2013). Photodynamic Therapy (PDT): PDT Mechanisms. Clin. Endosc..

